# Seasonal variations in microbial and physicochemical parameters of Nile river water in Giza, Egypt: comparison of IDEXX and conventional methods

**DOI:** 10.1186/s12896-025-01076-z

**Published:** 2025-12-08

**Authors:** Ahmed S. Saied, Adel A. Mousa, Abdullah M. Abdo

**Affiliations:** Faculty of Science, Botany and Microbiology Department, Al- Azhar University, Nasr City, Cairo 11884 Egypt

**Keywords:** Nile river, Microbial contamination, Water quality, Seasonal variation, Physicochemical, IDEXX

## Abstract

The Nile River is an essential water source for millions of Egyptians, but it is increasingly threatened by microbial and chemical contamination linked to urbanization and seasonal changes. This study investigated seasonal variations in microbial pollution and physicochemical characteristics of Nile water at four sites in Giza Governorate using culture-based methods and the IDEXX Colilert-18 system. The highest total coliform counts occurred in summer, reaching 21,462 MPN/100 ml (IDEXX) and 17,748 CFU/100 ml (traditional methods). Fecal coliforms ranged from 995 CFU/100 ml in summer to 463 CFU/100 ml in winter, while fecal *Streptococcus* peaked in autumn. *Pseudomonas aeruginosa* was slightly elevated in summer (1,140 CFU/100 ml), and traces of *Salmonella* and *Staphylococcus* were also detected. IDEXX consistently yielded 8–10% higher counts than traditional methods, reflecting greater sensitivity. Seasonal fluctuations were also observed in physicochemical parameters, including temperature (18.6–31.6 °C), pH (8.02–8.26), turbidity (3.89–5.74 NTU), total dissolved solids (205–279 mg/L), and electrical conductivity (310–435 µS/cm). Biological and chemical oxygen demands were highest in summer. Significant correlations (*p* < 0.05) were found between microbial counts and factors such as temperature and turbidity. These findings highlight elevated contamination risks during summer and underscore the enhanced detection ability of IDEXX. Significant correlations (*p* < 0.05, ANOVA) were found between microbial counts and temperature, turbidity, and BOD levels. These results emphasize the need for enhanced surveillance to protect public health, particularly during the summer when contamination risk peaks.

**Supplementary Information:**

The online version contains supplementary material available at 10.1186/s12896-025-01076-z.

## Introduction

The Nile River is Egypt’s primary freshwater source, sustaining over 100 million people through its use in drinking water supply, agriculture, and industry. As the longest river in the world, it plays a critical role in Egypt’s economy and public health [1]. However, rapid urbanization, agricultural expansion, and industrial discharge have led to progressive deterioration of water quality, with microbial contamination emerging as a major threat [2, 3]. Similar challenges have been reported in other major rivers worldwide, such as the Ganges in India, the Yangtze in China, and the Niger in West Africa, where untreated wastewater, industrial effluents, and agricultural runoff drive widespread bacterial pollution [4–6]. These global comparisons underscore the urgency of robust monitoring strategies for rivers serving as lifelines in developing regions [7].

Bacterial contamination of surface waters is strongly associated with public health risks, as pathogenic species are often linked to gastrointestinal diseases, typhoid fever, and other waterborne infections. In the Nile, documented contaminants include total coliforms, fecal coliforms, *Escherichia coli*, *Salmonella spp.*, *Staphylococcus aureus*, and *Pseudomonas aeruginosa* and *Klebsiella Pneumoniae* [8–11]. Several studies report bacterial counts exceeding permissible limits, especially near densely populated urban centers such as Giza and Cairo, where untreated sewage and industrial effluents are discharged directly into the river [5, 12]. Seasonal fluctuations further influence microbial levels: higher summer counts are attributed to elevated temperatures and reduced water flow, while winter generally shows lower values due to increased turbulence and dilution effects [13–15]. Seasonal variations significantly influence bacterial contamination levels in the Nile River. During the summer months, bacterial counts tend to be highest due to elevated temperatures, which promote microbial growth, and reduced water flow, which limits the natural dilution of pollutants [16]. In contrast, bacterial concentrations tend to decline in winter due to lower temperatures and increased water turbulence, which helps disperse contaminants. Seasonal fluctuations also result from changing precipitation patterns, which can either flush contaminants into the river during heavy rainfall or lead to stagnation during dry periods. These seasonal dynamics highlight the need for year-round monitoring to assess water quality effectively and identify periods of heightened risk [17].

Reliable detection of microbial contamination is central to safeguarding water quality. Traditional culture-based methods, including selective media and biochemical assays, remain widely used but require long incubation times (24–48 h), cannot detect viable but non-culturable (VBNC) bacteria, and demand laboratory infrastructure and skilled personnel [18–20]. These limitations highlight the need for faster and more sensitive alternatives. The IDEXX Colilert-18 system employs enzyme-substrate reactions to detect coliforms and *E. coli* within 18 h, offering greater sensitivity and efficiency than traditional culture approaches [21, 22]. Comparative studies confirm that IDEXX achieves higher accuracy in routine water assessments, making it a valuable complement to established techniques [23, 24].

This study hypothesizes that the IDEXX Colilert-18 system yields higher microbial detection rates than traditional methods when applied to Nile River water in Giza Governorate under varying seasonal and physicochemical conditions. The study further evaluates seasonal variations in bacterial populations and examines their correlations with physicochemical parameters, including pH, turbidity, total dissolved solids (TDS), and nutrient loads. By integrating modern and traditional microbiological approaches, this work provides evidence-based insights into seasonal and spatial contamination patterns, supporting strategies to enhance water quality monitoring and mitigate public health risks associated with Egypt’s primary freshwater resource.

## Materials and methods

### Materials

For microbiological analyses, standard selective and non-selective media were used, summarized in Supplementary Table S1. General-purpose media included Plate Count Agar (Oxoid, UK) for total heterotrophic bacteria. Selective media were employed for indicator and pathogenic groups: Endo-type agar for total coliforms, m-FC agar for fecal coliforms and *Escherichia coli*, Slanetz and Bartley medium for *Enterococcus spp.*, Compact Dry *Salmonella* plates for *Salmonella spp.*, *Pseudomonas* CFC/Kings B agar for *Pseudomonas aeruginosa*, and Compact Dry X-SA plates for *Staphylococcus aureus*.

To ensure quality control, certified reference strains (*E. coli* ATCC 25922, *Salmonella enterica* serovar Typhimurium ATCC 14028, *Enterococcus faecalis* ATCC 29212, *Pseudomonas aeruginosa* ATCC 27853, and *Staphylococcus aureus* ATCC 25923) were obtained from the American Type Culture Collection (ATCC, USA). These were used to validate selective media performance and biochemical tests.

For rapid detection and quantification of coliforms, the Colilert-18 reagent with Quanti-Tray/2000 system (IDEXX Laboratories, USA) was applied according to the manufacturer’s protocol.

For physicochemical analysis, on-site parameters (temperature, pH, turbidity, TDS, EC, DO, BOD₅, COD) were measured using portable multiparameter instruments following APHA (2017) standards. To assess heavy metals, samples were acidified with ultrapure HNO₃ immediately after collection, and trace metals (Pb, Cd, Hg, Fe, Cu, Zn, Mn) were quantified using Flame Atomic Absorption Spectrophotometry (PerkinElmer Analyst 400) in accordance with EPA Method 200.9.

All procedures were performed in triplicate with positive and negative controls. Data were tested for normality before applying ANOVA and correlation analyses to ensure the validity of statistical assumptions.

### Study area and sample collection

**Fig. 1:**
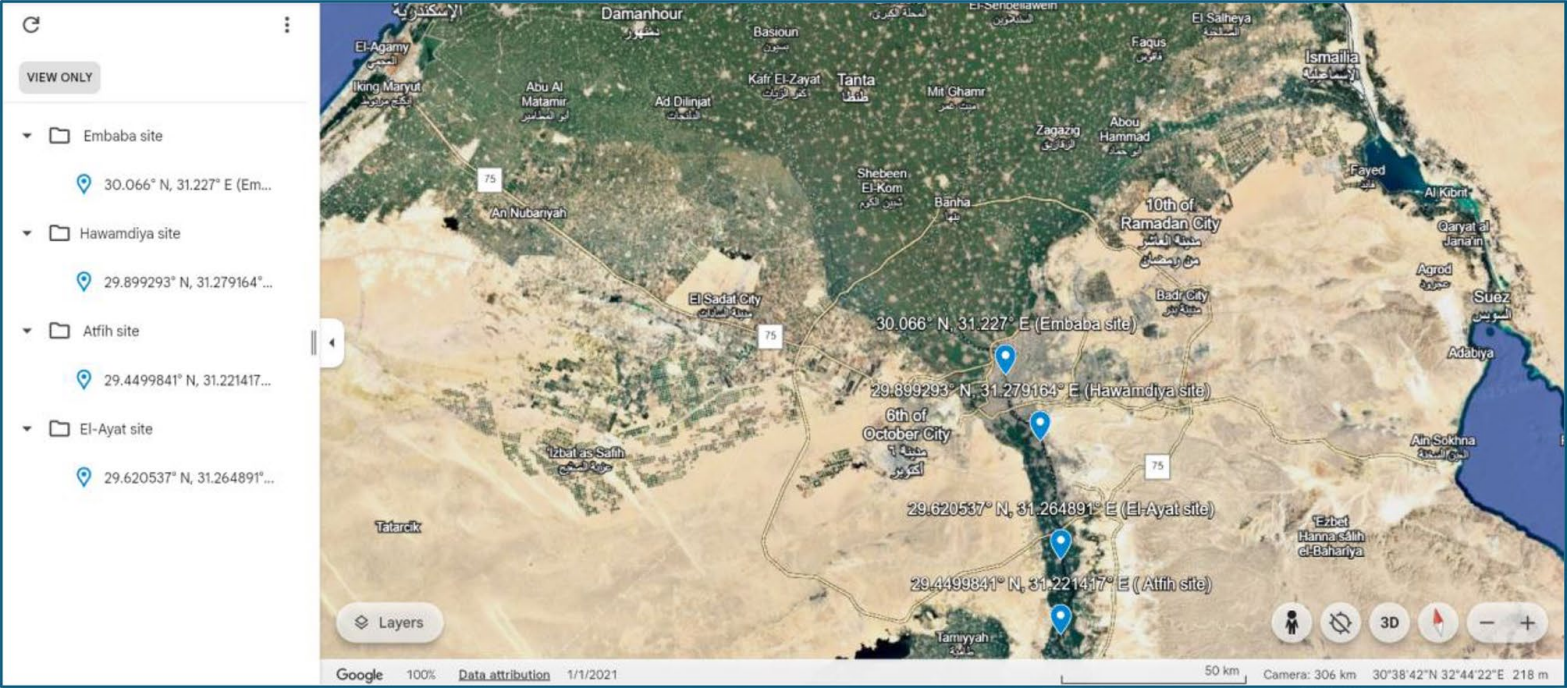
Satellite view of the Nile River near Giza Governorate, Egypt, as captured from Google Earth [25]. Sampling sites: Embaba (urban), Hawamdiya (peri-urban/industrial), Atfih (semi-rural), and El-Ayat (rural)

This study was conducted from March 2022 to February 2023 in Giza Governorate, Egypt, at four sites (Embaba, Hawamdiya, Atfih, and El-Ayat), representing urban, peri-urban/industrial, agricultural, and rural environments. Embaba (30.066° N, 31.227° E) is a densely populated urban area influenced by sewage and industrial discharges; Hawamdiya (29.899° N, 31.279° E) is peri-urban with mixed industrial and agricultural inputs; Atfih (29.449° N, 31.221° E) is semi-rural, dominated by irrigation return flows; and El-Ayat (29.621° N, 31.265° E) is rural with minimal industrial impact (Fig. 1). Water sampling was performed seasonally (spring, summer, autumn, winter) following APHA (2017) protocols. At each site, 1 L of raw water samples were collected in sterile polyethylene bottles at 20 cm below the surface and 1 m below. Treated water samples (120 ml) were collected in sterile glass bottles containing 0.1 ml of 3% sodium thiosulfate (Na₂S₂O₃) to neutralize residual chlorine. Each site was sampled in triplicate per season (*n* = 3 per site per season), totaling 48 samples. All samples were transported in insulated iceboxes at 4 °C and processed within six hours to prevent microbial or chemical alterations.

### Physicochemical analysis

Field parameters, including temperature, pH, turbidity, total dissolved solids (TDS), and electrical conductivity (EC), were measured on-site using calibrated portable instruments (CONSORT C535 multiparameter analyzer and Hach 2100 N turbidimeter) immediately after sampling, following APHA (2017) protocols. Dissolved oxygen (DO) was measured with a YSI ProODO optical meter, while biological oxygen demand (BOD₅) was determined using the standard five-day incubation method at 20 °C. Chemical oxygen demand (COD) was analyzed by the closed reflux titrimetric method, both in accordance with APHA (2017) [26]. For trace metal analysis, samples were acidified to pH < 2 with ultrapure nitric acid (HNO₃) immediately after collection and stored at 4 °C until analysis. Concentrations of Pb, Cd, Hg, Fe, Cu, Zn, and Mn were quantified using Flame Atomic Absorption Spectrophotometry (PerkinElmer Analyst 400) according to EPA Method 200.9. Detection limits (LOD) for heavy metals ranged from 0.002 to 0.05 mg/L, depending on element sensitivity.

All measurements were conducted in triplicate, with routine instrument calibration checks and inclusion of quality control standards in each analytical run to ensure data reliability. Samples were collected seasonally (spring, summer, autumn, and winter) from four sites along the Nile River and analyzed to assess temporal and spatial variations in physicochemical characteristics, which are critical for understanding microbial contamination dynamics and overall water quality [27].

### Microbiological analysis

Water samples (100 ml) were processed using membrane filtration and pour plate methods following APHA (2017) guidelines. Bacterial growth was enumerated as colony-forming units per 100 ml (CFU/100 ml). Selective and non-selective media were summarized in Supplementary Table S1 to avoid repetition in the text. Indicator bacteria were quantified using Endo-type agar for total coliforms, m-FC agar for fecal coliforms and *Escherichia coli*, and Slanetz & Bartley medium for *Enterococcus spp.* Pathogenic bacteria were detected on CompactDry *Salmonella* (for *Salmonella spp.*), Pseudomonas CFC/Kings B agar (for *Pseudomonas aeruginosa*), and CompactDry X-SA (for *Staphylococcus aureus*). Reference strains (*E. coli* ATCC 25922, *Salmonella enterica* serovar Typhimurium ATCC 14028, *Enterococcus faecalis* ATCC 29212, *Pseudomonas aeruginosa* ATCC 27853, and *Staphylococcus aureus* ATCC 25923) were obtained from the American Type Culture Collection (ATCC, USA) to validate media performance and biochemical tests. Biochemical confirmation of isolates included oxidase, catalase, and IMViC tests performed under standardized conditions. For rapid detection, the IDEXX Colilert-18 with Quanti-Tray/2000 system (IDEXX, USA) was used for total coliforms and *E. coli*, providing results within 18 h based on colorimetric and fluorescence reactions. All microbiological assays were conducted in triplicate with appropriate positive and negative controls to ensure accuracy and reproducibility [23, 28].

### Quality control and data analysis

All microbiological assays were performed in triplicate, incorporating positive controls (*Escherichia coli* ATCC 25922, *Salmonella enterica* ATCC 14028, *Enterococcus faecalis* ATCC 29212, *Pseudomonas aeruginosa* ATCC 27853, and *Staphylococcus aureus* ATCC 25923) and sterile distilled water as a negative control. This ensured the reliability of selective media, biochemical tests, and IDEXX assays. Instrument calibration and quality control standards were routinely applied for physicochemical measurements. Data were statistically analyzed using IBM SPSS Statistics v26 (IBM Corp., USA). One-way ANOVA was used to assess seasonal and spatial variations, with a significance threshold of *p* < 0.05. Triplicate readings were averaged before statistical analysis. Data normality and homogeneity of variance were confirmed using Shapiro–Wilk and Levene’s tests before applying one-way ANOVA. This approach ensured that statistical conclusions were valid, reproducible, and consistent with standard environmental monitoring practices [29].

## Results and discussion

### Seasonal microbial contamination patterns

Significant seasonal and spatial variations in microbial contamination were detected across the Nile River sites in Giza Governorate (one-way ANOVA, *p* < 0.05). Total bacterial, fecal indicator, and pathogenic species exhibited distinct seasonal distributions driven by temperature, turbidity, and organic load.

**Fig. 2:**
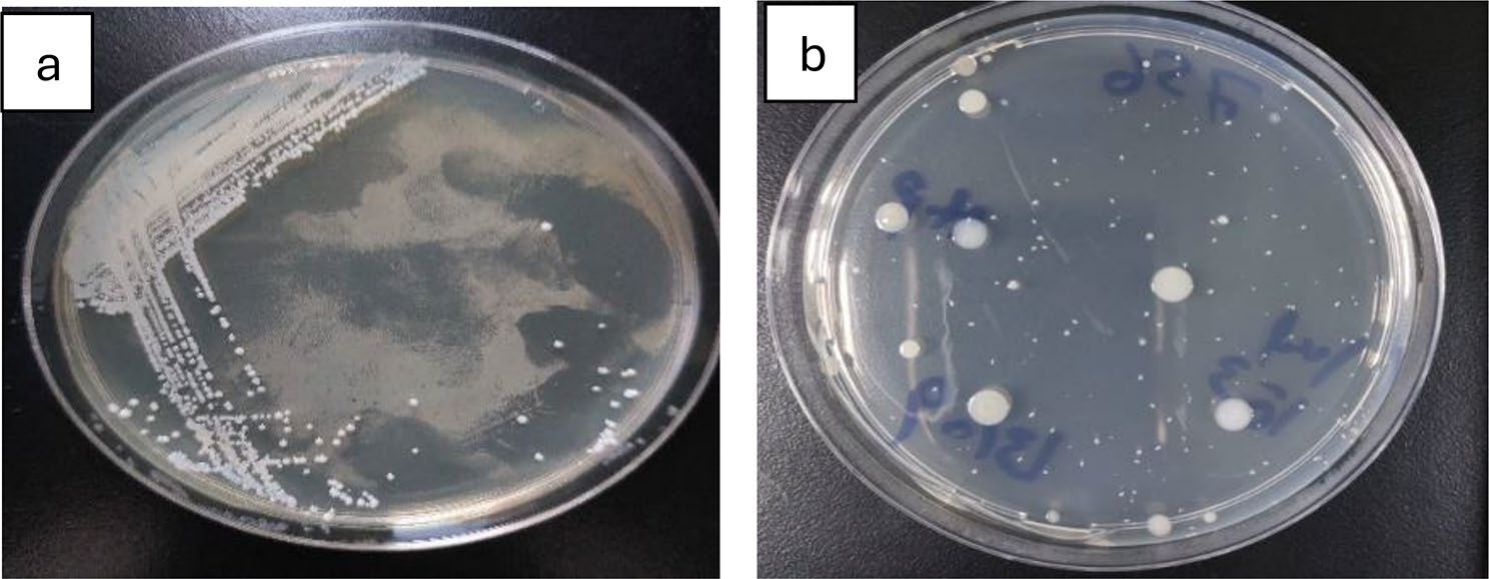
Estimation of heterotrophic bacteria using the Pour Plate Method, representative images from triplicate analyses. (**a**) Plate count agar with streak; (**b**) plate count agar showing bacterial colonies

Overall, microbial counts peaked in summer and reached their lowest in winter. The highest total coliform levels were recorded in summer (21,462 ± 1,275 MPN/100 mL, IDEXX; 17,748 ± 1,103 CFU/100 mL, culture), declining in winter to 1,300–1,769 CFU/100 mL. These seasonal differences were statistically significant (*p* < 0.05) and correlated with temperature (*r* = 0.68, *p* < 0.01) and turbidity (*r* = 0.54, *p* < 0.05). Higher summer values reflect elevated microbial metabolism under warm, less-diluted conditions, consistent with observations in other tropical rivers such as the Ganges and Niger [13, 30, 31]. Morphologically, heterotrophic colonies were circular, opaque, smooth, and white to off-white (Fig. 2).

**Fig. 3:**
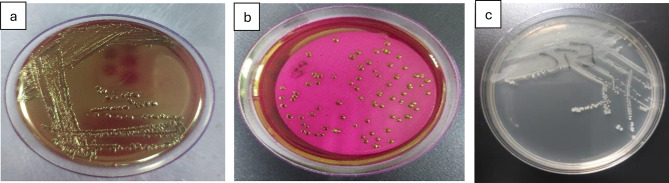
Detection and enumeration of total coliform bacteria using the Membrane Filter Technique, representative images from triplicate analyses. (**a**) m-endo les agar with streaking; (**b**) m-endo les agar showing coliform colonies. (**c**) Plate count agar with streak

Total coliforms followed the same pattern, reaching 21,462 MPN/100 mL (IDEXX; log ≈ 4.33) versus 17,748 CFU/100 mL (culture; log ≈ 4.25) in summer, with the lowest winter values around log 3.1–3.3 (Table 1; Fig. 10). On Endo-type agar, colonies showed the metallic sheen typical of *E. coli* (Fig. 3). IDEXX values were 8–10% higher (*p* = 0.032, df = 3), confirming its greater sensitivity for VBNC detection [32].

**Fig. 4:**
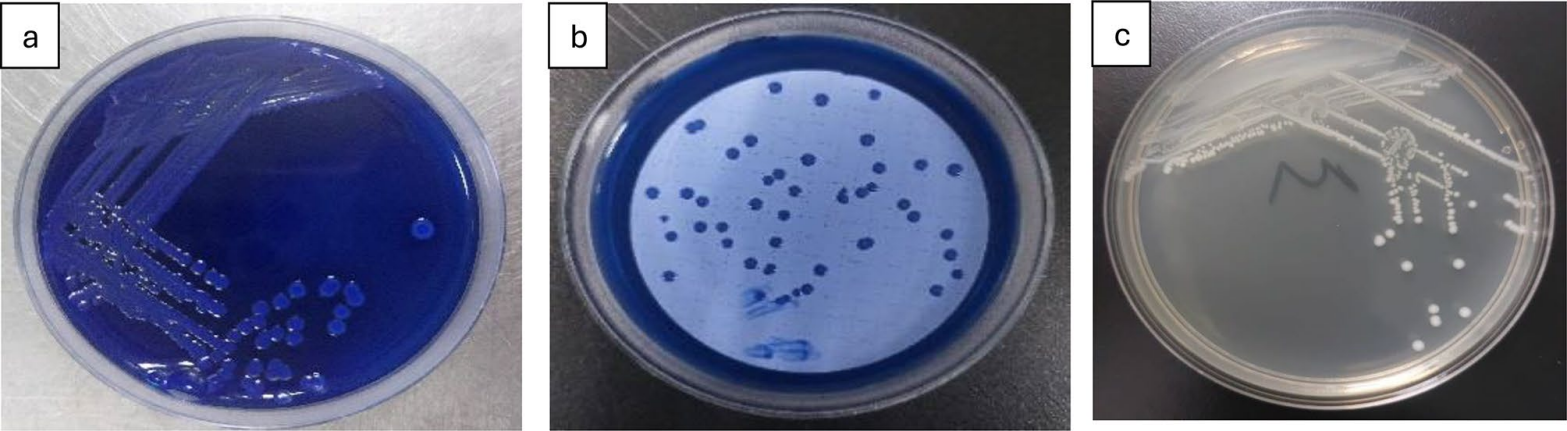
Detection and enumeration of thermotolerant (fecal) coliform bacteria using the Membrane Filter Technique, representative images from triplicate analyses. (**a**) MFC agar with streaking; (**b**) MFC agar showing fecal coliform colonies. (**c**) Plate count agar with streak

**Fig. 5:**
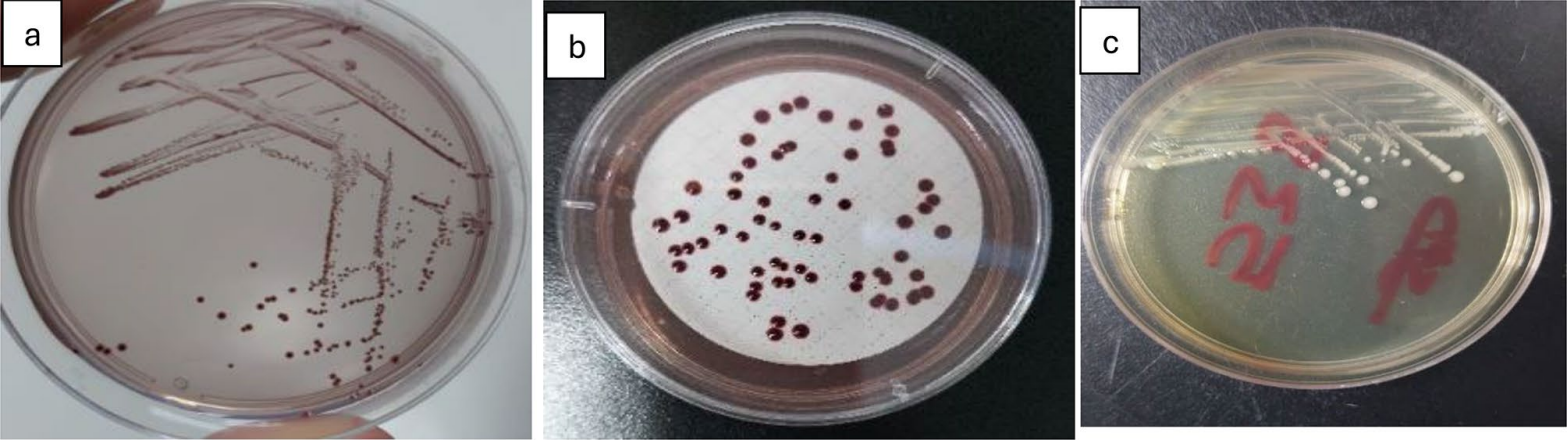
Detection and enumeration of fecal *Enterococcus/Streptococcus* groups using the Membrane Filter Technique, representative images from triplicate analyses. (**a**) Slanetz and Bartley medium with streaking; (**b**) Slanetz and Bartley media showing Fecal *Enterococcus* colonies; (**c**) plate count agar with streak

Fecal coliforms ranged from 995 CFU/100 mL in summer (log ≈ 2.99) to 463 CFU/100 mL in winter (log ≈ 2.67) (*p* = 0.041) (Fig. 10). On m-FC agar (44.5 °C), colonies appeared blue due to acid production (Fig. 4). Fecal streptococci peaked in autumn (113 CFU/100 mL, log ≈ 2.05) and declined in winter (23 CFU/100 mL, log ≈ 1.36), with Hawamdiya >Atfih (*p* < 0.01) (Fig. 10). Colonies on Slanetz & Bartley medium were reddish-maroon and dry (Fig. 5), consistent with *Enterococcus faecalis* morphology [31, 33].

**Fig. 6:**
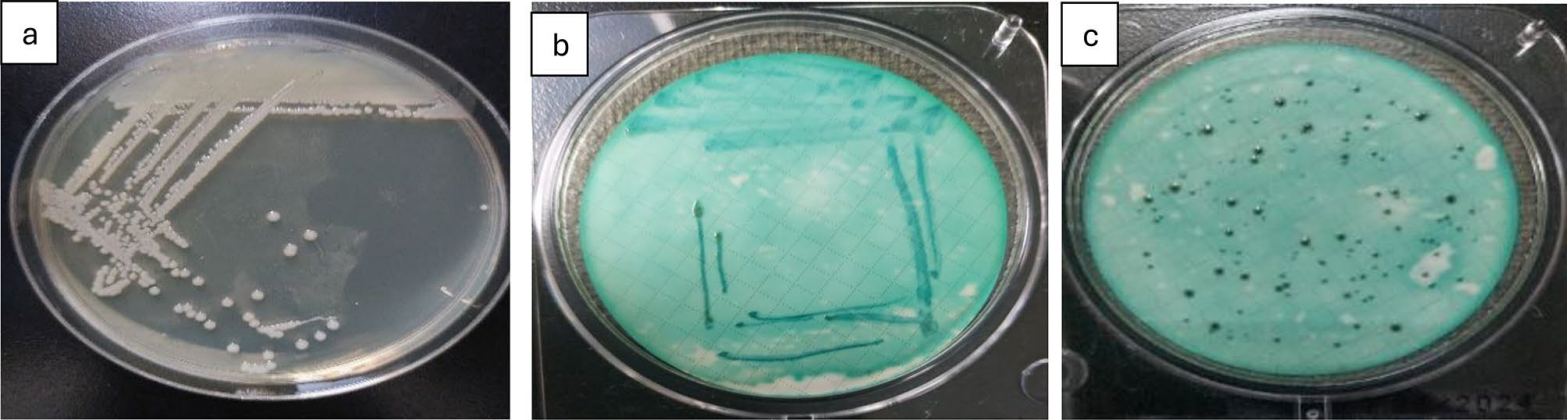
Detection and enumeration of *Salmonella* using the Membrane Filter Technique, representative images from triplicate analyses. (**a**) CompactDry Salmonella plate with streaking; (**b**) CompactDry *Salmonella* plate showing *Salmonella* colonies

Pathogenic bacteria were detected year-round but varied by season. – *Salmonella spp.* Peaked in autumn (75 CFU/100 mL, log ≈ 1.88) and summer (63 CFU/100 mL, log ≈ 1.80), reflecting runoff and livestock waste inputs [34]. Typical colonies on CompactDry plates were violet with dark centers (Fig. 6). Such dual coloration and dark cores are characteristic diagnostic markers [35].

**Fig. 7:**
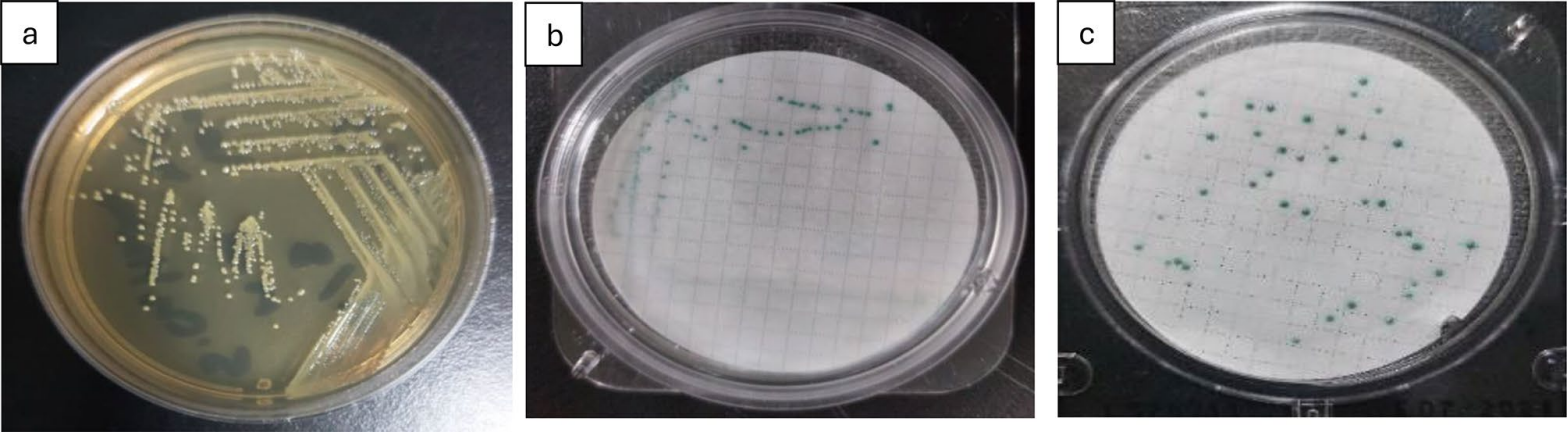
Detection and enumeration of Staphylococcus using the Membrane Filter Technique, representative images from triplicate analyses. (**a**) CompactDry Staph plate with streaking; (**b**) CompactDry Staph plate showing *Staphylococcus colonies*

*Staphylococcus aureus* was most abundant in summer (92 CFU/100 mL, log ≈ 1.96) and least in winter (25 CFU/100 mL, log ≈ 1.40) (Fig. 10), consistent with its thermotolerance and chlorine resistance [36, 37]. Golden-yellow colonies on X-SA plates confirmed *S. aureus* identity (Fig. 7).

**Fig. 8:**
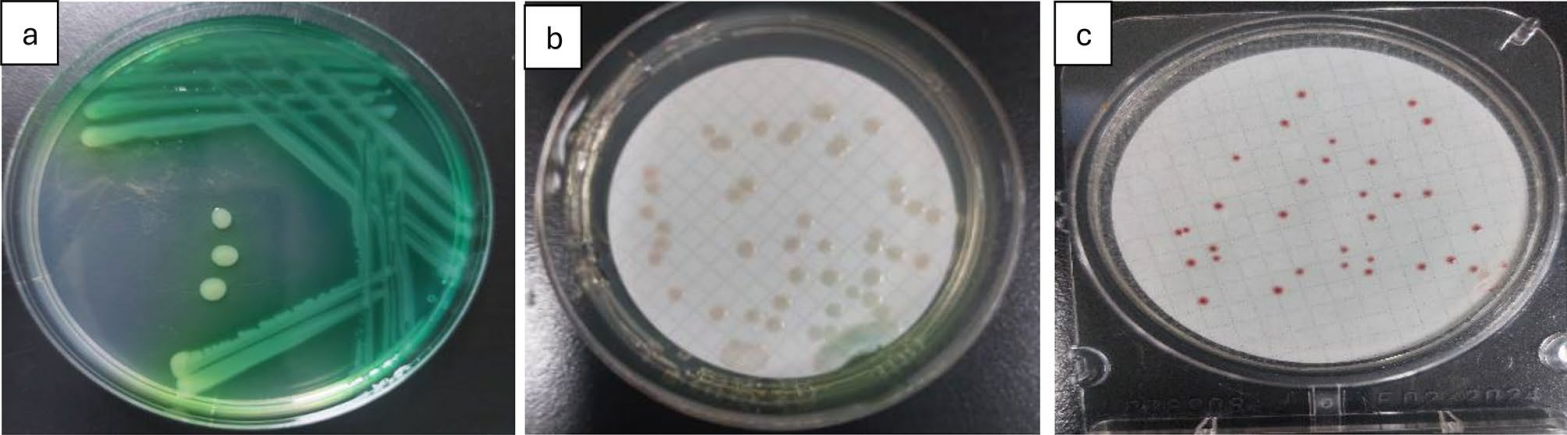
Detection and enumeration of Pseudomonas aeruginosa using the Membrane Filter Technique, representative images from triplicate analyses. (**a**) King B agar with streaking; (**b**) CFC/CN agar showing Pseudomonas colonies; (**c**) CompactDry Pseudomonas plate showing *Pseudomonas aeruginosa* colonies

*Pseudomonas aeruginosa* peaked in spring (1,933 CFU/100 mL, log ≈ 3.29) and remained elevated in summer (1,140 CFU/100 mL, log ≈ 3.06) (Fig. 10). Its persistence indicates adaptation to nutrient-rich, low-oxygen environments [38]; bluish-green pigment on King’s B agar and flat, irregular colonies on CFC agar verified species identity (Fig. 8). Pigments such as pyocyanin and pyoverdine serve as reliable markers for identification in clinical and environmental samples.

In summary, summer conditions and urban influence markedly enhanced microbial loads, while IDEXX consistently captured higher counts due to VBNC cell detection. These findings underscore the combined effects of temperature, turbidity, and anthropogenic inputs on seasonal water quality in the Nile River.

### Seasonal and spatial trends

Overall, pathogenic bacterial levels exhibited clear seasonal and site-dependent variation (*p* < 0.01), with urban Embaba consistently showing the highest contamination—highlighting the impact of untreated sewage discharge. Correlation analysis revealed positive associations between microbial counts and temperature (*r* = 0.68, *p* < 0.01) and turbidity (*r* = 0.54, *p* < 0.05), while pH had no significant effect. These findings parallel reports from tropical rivers such as the Ganges [31] and Niger [13], where warmer conditions and anthropogenic inputs elevate contamination risks. From a public health perspective, high microbial loads in summer, particularly in densely populated areas, raise the risk of gastrointestinal and enteric infections. This underscores the need for intensified monitoring and improved wastewater management during high-risk periods.

### IDEXX vs. traditional methods

**Fig. 9:**
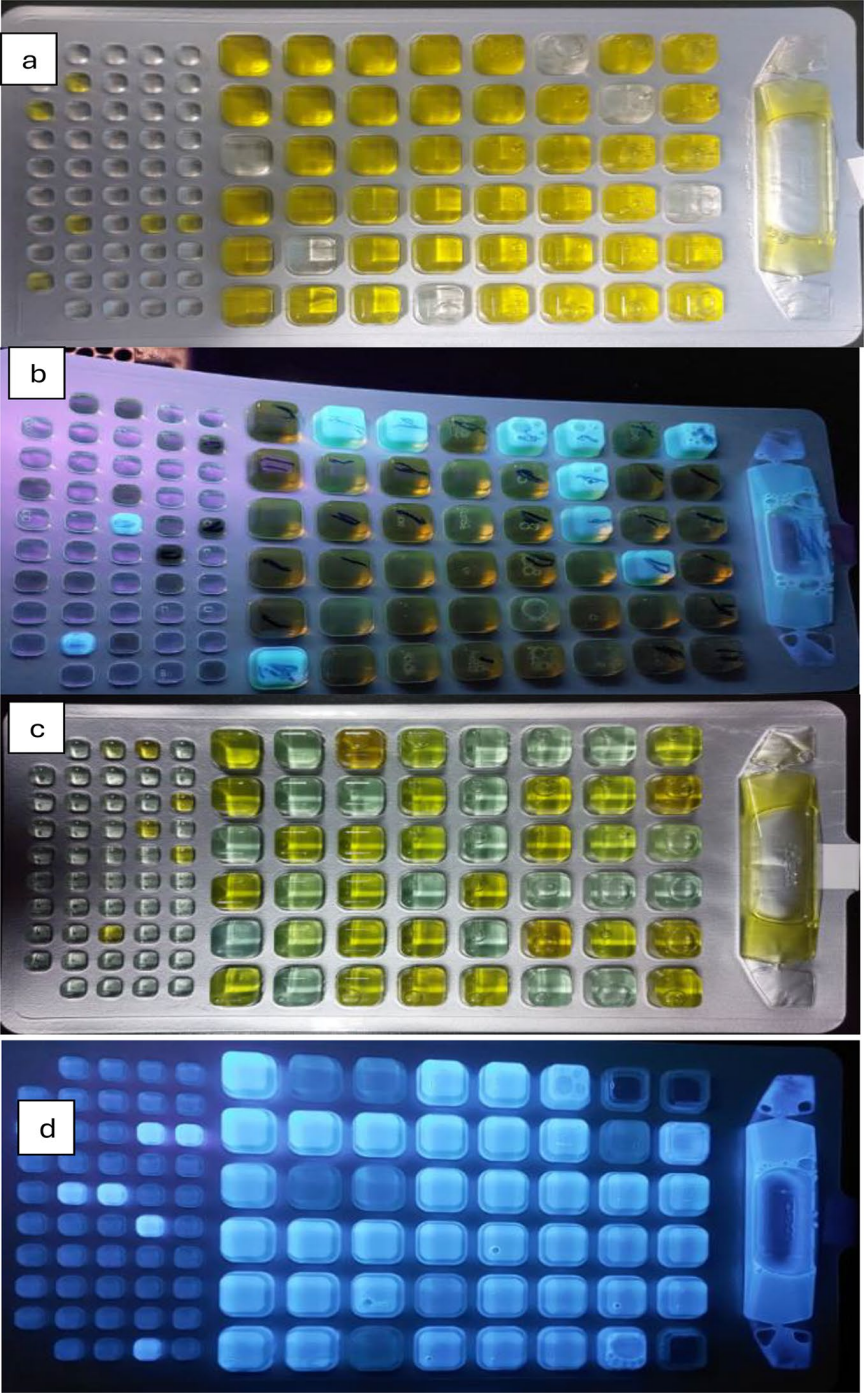
*Detection and enumeration of Bacterial contamination using the IDEXX Colilert-18 system Technique*, representative images from triplicate analyses. (**a**) Total coliform bacteria; (**b**) (fecal) coliform bacteria; (**c**) fecal Enterococcus/Streptococcus groups; (**d**) *Pseudomonas sp.* bacteria

**Fig. 10:**
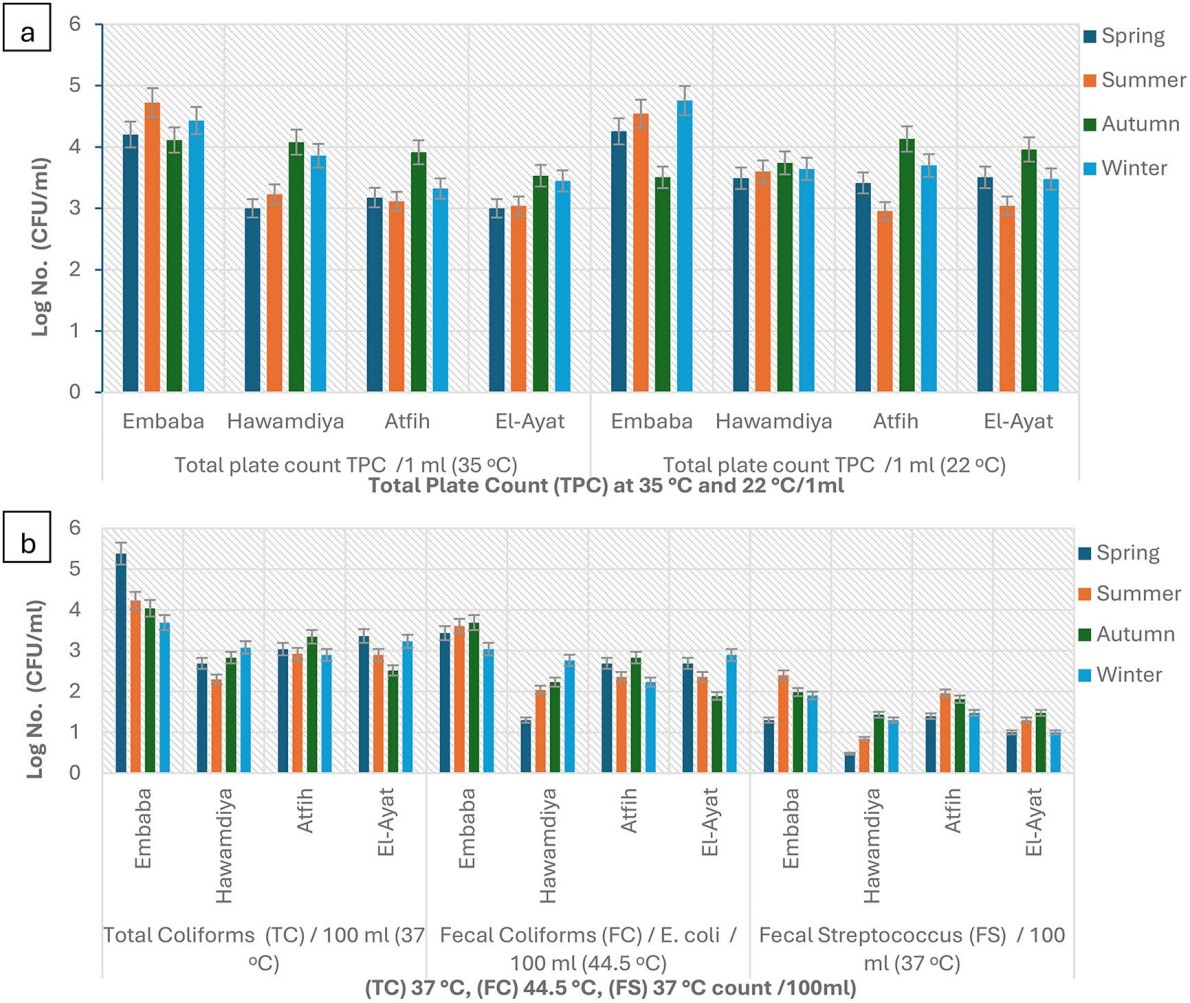

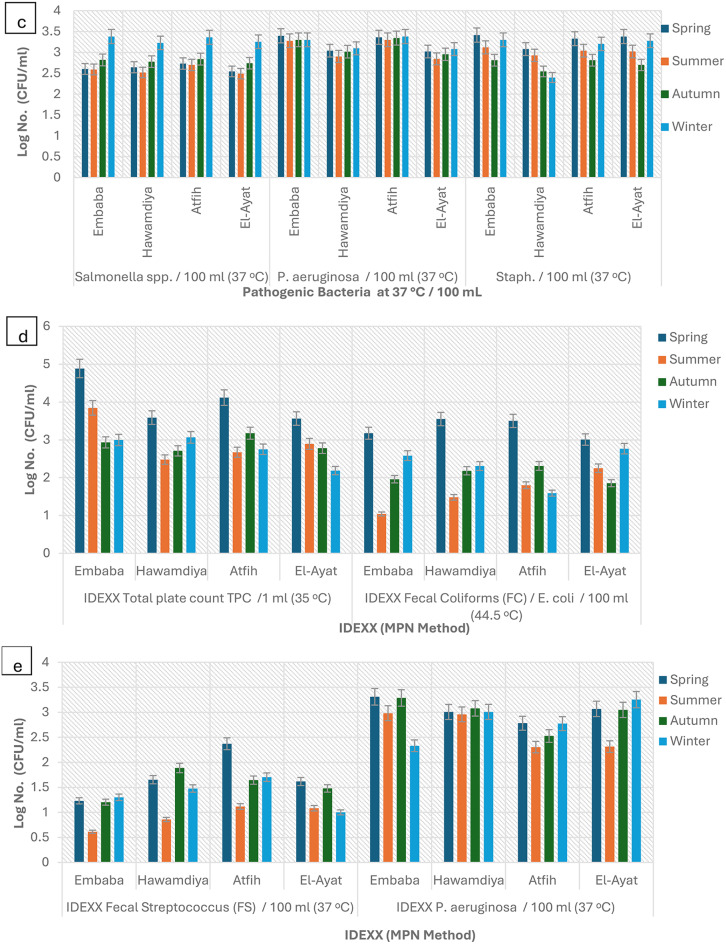
Seasonal Variation in Bacterial Contamination IDEXX Log. No. Counts of Nile River Water in Giza governorate. Values represent log-transformed means of triplicate measurements for each season and site. Error bars indicate mean ± standard deviation (SD; *n* = 3). Total Plate Count (TPC): ANOVA indicated significant seasonal variation in TPC values at both 35 °C and 22 °C (*p* < 0.05). Counts were generally higher in summer and winter, with Embaba showing consistently greater value compared to other sites (*p* < 0.01). Indicator Bacteria (Total Coliforms, Fecal Coliforms, Fecal Streptococcus): Significant differences were observed across both seasons and sites (*p* < 0.05). Total and Fecal Coliforms were significantly higher in summer and autumn, while Fecal Streptococcus peaked in summer (*p* < 0.01). Embaba and Hawamdiya recorded significantly higher counts compared to Atfih and El-Ayat. Pathogenic Bacteria (*Salmonella spp.*,* Pseudomonas aeruginosa*,* Staphylococcus spp.*): ANOVA confirmed site-dependent differences (*p* < 0.01), with Embaba consistently showing the highest counts. Seasonal effects were less pronounced but still significant for *P. aeruginosa* and Staphylococcus spp. (*p* < 0.05), with higher levels in summer and autumn compared to winter. Total Plate Count (TPC): ANOVA revealed significant seasonal differences in TPC values at 35 °C (*p* < 0.05). Counts were highest in Spring and Winter, while Summer and Autumn showed reduced levels. Across sites, Embaba consistently recorded the greatest values (*p* < 0.01), followed by Hawamdiya, whereas Atfih and El-Ayat showed significantly lower counts. Fecal Coliforms (*E. coli*): Significant variation was detected across both seasons and sites (*p* < 0.05). Spring samples exhibited the highest concentrations, with Autumn and Winter showing lower levels. Embaba and Hawamdiya recorded significantly higher counts compared to Atfih and El-Ayat (*p* < 0.01). Fecal Streptococcus (FS): ANOVA indicated strong seasonal variation (*p* < 0.05). Autumn recorded peak values, particularly at Hawamdiya and Atfih (*p* < 0.01), while Summer and Winter were consistently lower. Embaba also exhibited elevated FS counts relative to El-Ayat. Pseudomonas aeruginosa: Both site- and season-dependent differences were significant (*p* < 0.05). Embaba and Hawamdiya consistently showed the highest levels, while Atfih and El-Ayat remained lower. Seasonal effects were moderate but significant, with Autumn and Spring showing higher values than Summer and Winter (*p* < 0.05)

**Fig. 11:**
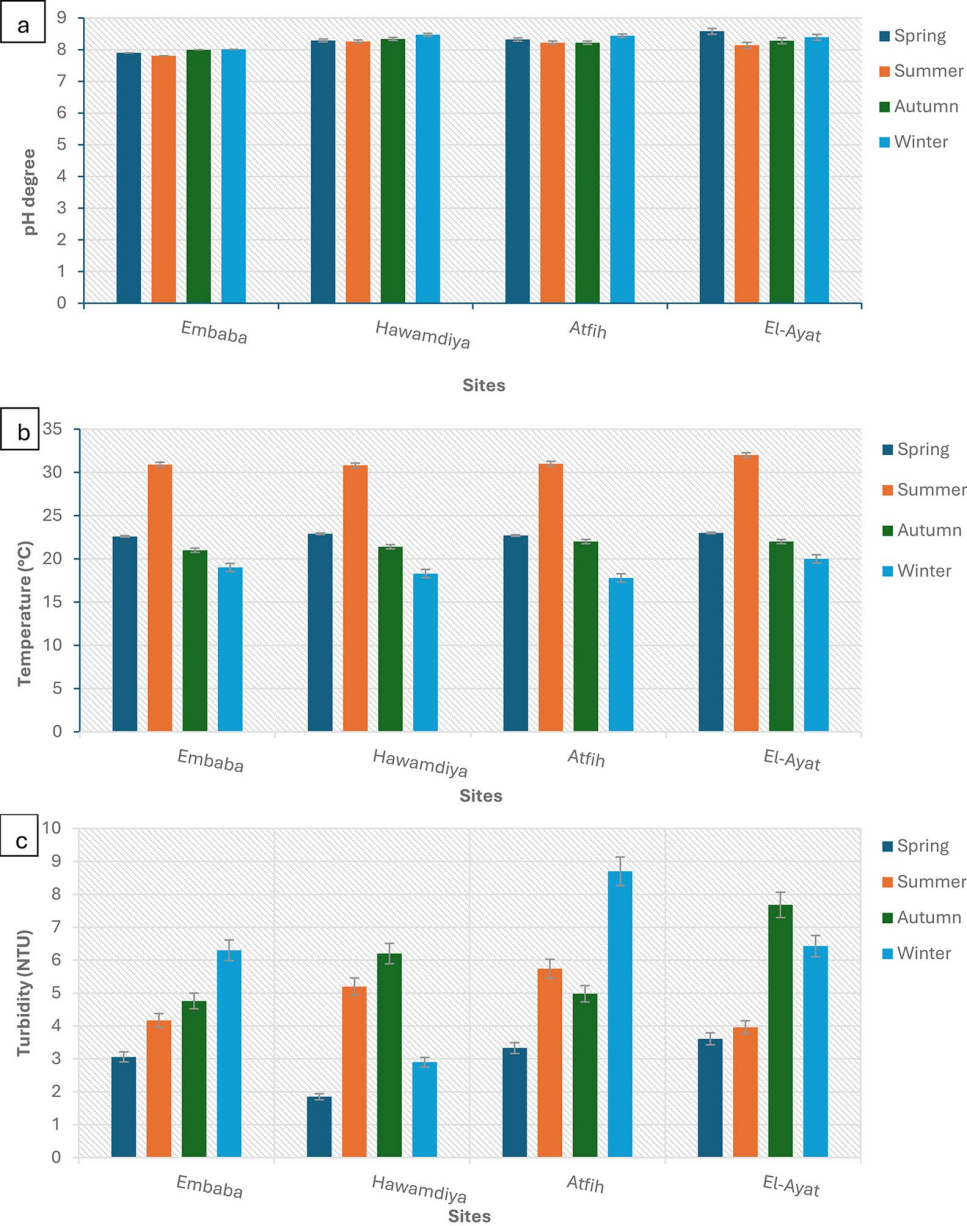

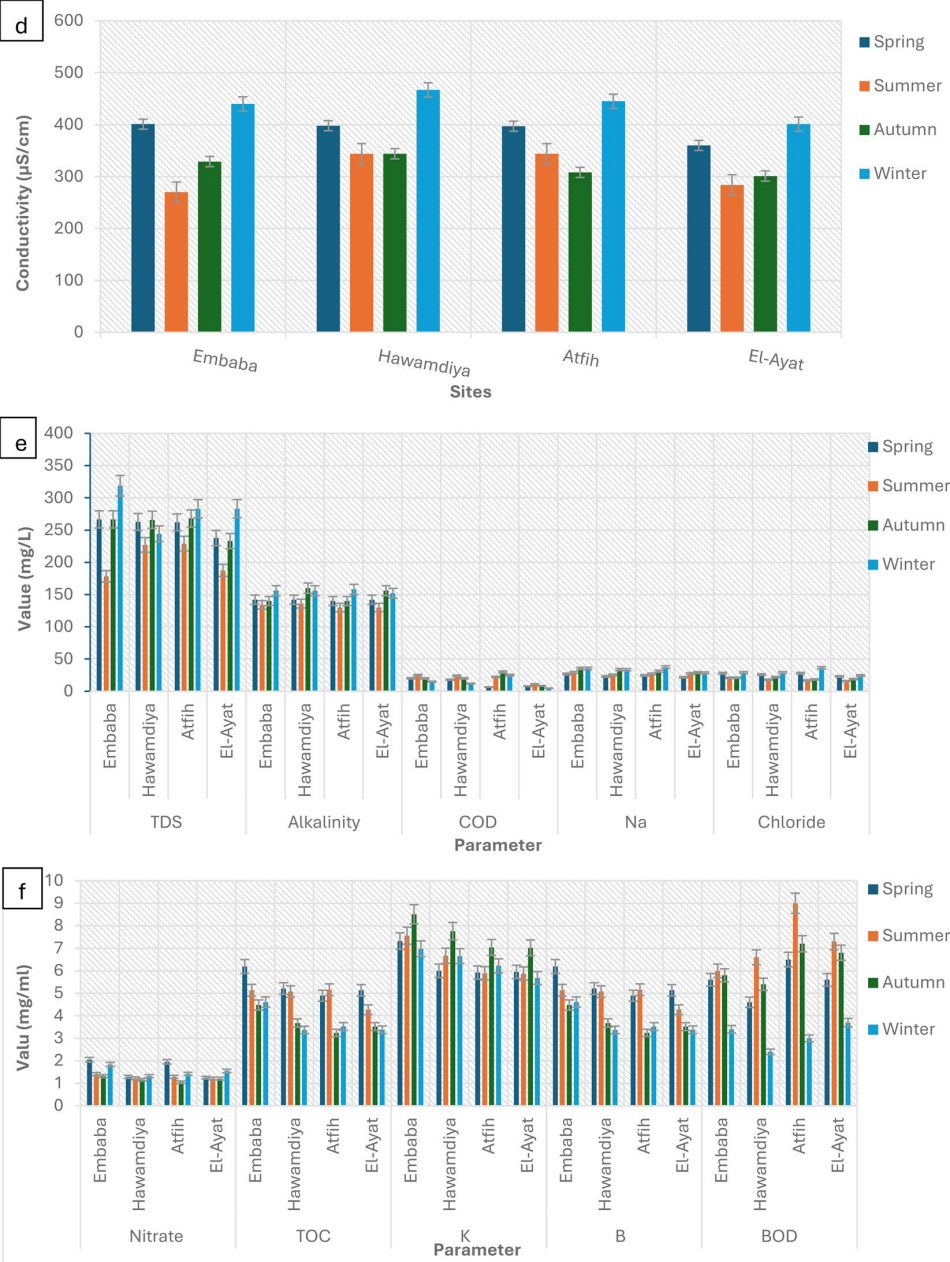
Seasonal Variation in Physicochemical Parameters of Nile River Water, Giza Governorate. Error bars represent mean ± SD (*n* = 3) for each sampling site and season. pH: ANOVA showed no significant seasonal variation in pH across all sites (*p* > 0.05). Values remained within a narrow alkaline range (7.5–8.4), with no site-specific differences. Temperature: Temperature varied significantly with season (*p* < 0.01), peaking in summer (30–32 °C) and lowest in winter (18–20 °C). Site effects were not significant (*p* > 0.05). Turbidity: Turbidity showed significant seasonal variation (*p* < 0.01), with higher values in winter and autumn compared to summer (*p* < 0.05). Atfih and El-Ayat recorded significantly greater turbidity compared to Hawamdiya (*p* < 0.05). Conductivity & TDS: Both conductivity and TDS exhibited significant seasonal variation (*p* < 0.05), with maximum values in winter and spring. Embaba consistently showed higher conductivity and TDS compared to other sites (*p* < 0.01). Alkalinity: Alkalinity showed moderate seasonal variation (*p* < 0.05), highest in summer and autumn. No significant site-dependent differences were detected (*p* > 0.05). COD, Na, and Chloride: COD varied significantly across seasons (*p* < 0.05), increasing in summer and autumn. Sodium and chloride levels did not differ significantly by season or site (*p* > 0.05). Nitrate: Nitrate concentrations were significantly affected by season (*p* < 0.01), with higher values in winter compared to other seasons. No site-dependent effect was observed. TOC and Potassium: TOC showed seasonal differences (*p* < 0.05), with elevated levels in spring and summer. Potassium varied significantly among sites (*p* < 0.05), with Embaba and Hawamdiya recording higher concentrations than Atfih (*p* < 0.01). Boron (B): Boron concentrations exhibited slight but significant seasonal differences (*p* < 0.05), with the highest values recorded in summer. Site variation was not significant (*p* > 0.05). BOD: BOD demonstrated both seasonal and site-dependent differences (*p* < 0.01). Higher levels were detected in summer and autumn, particularly at Embaba and El-Ayat compared to Atfih (*p* < 0.05)

The Colilert-18 system (IDEXX) consistently reported higher bacterial counts than traditional culture-based methods across all seasons (Table 1). This difference, averaging 8–10.5%, is attributed to IDEXX’s ability to detect viable but nonculturable (VBNC) bacteria that fail to grow on selective agar due to sublethal injury or nutrient limitations [39]. For example, in spring, total coliforms were measured at 21,462 MPN/100 ml by IDEXX compared to 20,759 CFU/100 ml by conventional culture. Colilert-18 results were visualized as yellow wells for total coliforms and fluorescent wells under UV light for *Escherichia coli* confirmation (Fig. 9). This method provided efficient enumeration using the Most Probable Number (MPN) approach, offering both sensitivity and ease of interpretation. When seasonal patterns were compared, IDEXX consistently detected higher log counts than culture methods (Figs. 10 and 11). These findings are in line with prior validation studies that demonstrated Colilert-18’s superior sensitivity, rapid turnaround time (18 h), and reliability in complex aquatic environments [39]. Collectively, these results reinforce the value of integrating rapid enzymatic assays such as IDEXX into water quality monitoring protocols, especially for early detection of contamination in surface waters subject to high seasonal and anthropogenic influences.

**Table 1:** Traditional culture vs. IDEXX difference in bacterial contamination counts of Nile river Water, Giza Governorate

Bacterial contamination counts	Season	Traditional culture	IDEXX (MPN Method)	% Difference
Total Coliforms (CFU/100 ml)	Spring	20759.25	21462.5	8.75%
	Summer	1748.25	1463	10.00%
	Autumn	4460	3904	10.00%
	Winter	1769.25	1300.025	10.53%
Fecal Coliforms (CFU/100 ml)	Spring	640	496.3	8.75%
	Summer	995	983.9	10.00%
	Autumn	1,365	869.175	10.00%
	Winter	530	463.6	10.53%
Fecal Streptococcus (CFU/100 ml)	Spring	19	14.275	8.75%
	Summer	54	39.825	10.00%
	Autumn	113	85.725	10.00%
	Winter	34.25	23.4	10.53%
*P. aeruginosa* (CFU/100 ml)	Spring	1,933	1287.6	8.75%
	Summer	1,140	1035.75	10.00%
	Autumn	495	433.85	10.00%
	Winter	1,350	1072.35	10.53%

Overall: There is no statistically significant difference (*p* > 0.05) between Traditional Culture and IDEXX (MPN Method) for most bacterial groups

Fecal *Streptococcus* shows a borderline difference (*p* ≈ 0.058), suggesting IDEXX may yield slightly lower values than traditional culture, but this is not conclusive at the 95% confidence level see Table S2

### Physicochemical parameters

Seasonal fluctuations in environmental conditions significantly influenced microbial contamination patterns in the Nile River (Fig. 11). Temperature: River water temperature varied between 18.6 °C in winter and 31.6 °C in summer. Elevated temperatures showed significant positive correlations (*p* < 0.05) with coliform and fecal bacterial counts, supporting the role of higher temperatures in accelerating microbial metabolism and reducing die-off rates [40]. pH: The river remained consistently slightly alkaline (8.02–8.26) throughout the study period, with no significant seasonal variation or detectable effect on bacterial levels [41]. Turbidity: Values ranged from 3.89 NTU in spring to 5.74 NTU in autumn. Increased turbidity likely enhanced microbial survival by shielding bacteria from UV penetration and providing suspended particles as attachment sites, thereby promoting persistence in the water column [42]. Total Dissolved Solids (TDS) and Conductivity: TDS values ranged between 205 and 279 mg/L, with higher levels in winter attributed to reduced dilution capacity. Electrical conductivity varied between 310 and 435 µS/cm, following the same trend, and reflected seasonal shifts in ionic strength [43]. Organic Load (BOD and COD): Biological oxygen demand (BOD) peaked in summer at 7.48 mg/L, reflecting elevated organic matter input, and declined to 3.13 mg/L in winter. Similarly, chemical oxygen demand (COD) reached 24.3 mg/L in summer before dropping to 16.75 mg/L in winter. These trends indicate stronger organic pollution during warmer months [44]. Heavy Metals: Lead (Pb), cadmium (Cd), and mercury (Hg) concentrations were largely below detection limits across sites and seasons. This suggests that microbial contamination was driven primarily by organic and fecal inputs rather than industrial metal pollution [45].

### Public health and management implications

The persistently high microbial counts, particularly during summer, represent a substantial public health risk, especially in urbanized areas such as Embaba, where untreated sewage and industrial effluents discharge directly into the Nile [46]. Given Egypt’s extensive dependence on the river for drinking water, irrigation, and recreation, these findings underscore the urgency of adopting stronger wastewater management and regulatory controls to curb point-source pollution.

Implementing rapid microbial detection technologies, such as the IDEXX Colilert-18 system, can markedly enhance early warning capacity for contamination events. Regular surveillance using IDEXX in parallel with traditional culture methods ensures comprehensive coverage, detecting both culturable and viable-but-non-culturable (VBNC) bacteria. Such dual monitoring systems provide a more accurate picture of water quality fluctuations and support timely interventions during high-risk periods, notably summer and post-rainfall events.

In addition to technological improvements, effective public communication and community engagement are essential. Public health advisories and educational programs promoting proper waste disposal and reduced household sewage discharge can substantially lower microbial loads entering the river.

Overall, a continuous, year-round monitoring framework that integrates advanced detection methods with conventional microbiological testing offers a robust approach to safeguarding the Nile’s water quality. This integrated strategy aligns with regional and international recommendations for sustainable river basin management and public health protection [47].

## Conclusion

This study provides the first systematic comparison of microbial contamination in the Nile River in Giza, demonstrating clear seasonal patterns with peak bacterial loads in summer. The IDEXX Colilert-18 system detected 8–10% more contamination than conventional methods, confirming its higher sensitivity and value for monitoring programs. Physicochemical drivers such as temperature, turbidity, and BOD were closely linked to microbial levels, while minimal heavy metal detection indicates fecal and organic pollution as the main sources. Elevated contamination in urban and peri-urban stretches highlights urgent public health risks, including increased exposure to waterborne diseases. These findings underscore the need for continuous year-round monitoring, improved wastewater infrastructure, and targeted interventions to protect community health. Safeguarding Nile water quality is essential for Egypt’s environmental sustainability and population health resilience. Future work should integrate molecular or qPCR-based methods for pathogen confirmation to complement IDEXX-based surveillance.

## Supplementary Information

Below is the link to the electronic supplementary material.

Supplementary Material 1

## Data Availability

The datasets generated during and/or analyzed during the current study are available from the corresponding author on reasonable request.
