# Supplementary material for: Seasonal variations in microbial and physicochemical parameters of Nile river water in Giza, Egypt: comparison of IDEXX and conventional methods
Source: BMC Biotechnol. 2025 Dec 8;25:138. doi: 10.1186/s12896-025-01076-z (PMC12690835; doi:10.1186/s12896-025-01076-z)
Supplement: Supplementary file 1 — Supplementary Material 1 [file 12896_2025_1076_MOESM1_ESM.docx]

**Table S1**. Selective and non-selective media used for microbial analysis of Nile River water samples.

| **Medium / System** | **Target Organism(s)** | **Incubation Conditions** |
| --- | --- | --- |
| Plate Count Agar (PCA, Oxoid, UK) | Total heterotrophic bacteria | 35 °C for 48 h or 22 °C for 5–7 d |
| Endo-type agar (Merck, Germany) | Total coliforms | 35 °C for 24 h |
| m-FC agar (Difco, BD, USA) | Fecal coliforms, *Escherichia coli* | 44.5 °C for 24 h |
| Slanetz & Bartley agar (Oxoid, UK) | *Enterococcus spp.* (fecal streptococci) | 35 °C for 48 h |
| CompactDry Salmonella (Nissui, Japan) | *Salmonella spp.* | 35–37 °C for 20–24 h (after BPW pre-enrichment) |
| Pseudomonas CFC Agar (Oxoid, UK) & King’s B Agar (Himedia, India) | *Pseudomonas aeruginosa* | 37 °C for 48 h |
| CompactDry Staphylococcus (X-SA, Nissui, Japan) | *Staphylococcus aureus* | 35 °C for 24 ± 2 h |
| IDEXX Colilert-18 with Quanti-Tray/2000 (IDEXX, USA) | Total coliforms, *E. coli* | 35 °C for 18 h (yellow/fluorescence readout) |

**Table S2.** Paired t-test Analysis of Traditional Culture vs. IDEXX (MPN Method) for Bacterial Contamination Counts in Nile River Water (Giza Governorate).

| **Bacterial Group** | **t-statistic** | **p-value** | **Interpretation** |
| --- | --- | --- | --- |
| Total Coliforms | 0.52 | 0.637 | No significant difference |
| Fecal Coliforms | 1.65 | 0.199 | No significant difference |
| Fecal Streptococcus | 2.99 | 0.058 | Borderline (close to significance at 0.05) |
| *P. aeruginosa* | 2.05 | 0.133 | No significant difference |

Overall: There is no statistically significant difference (p > 0.05) between Traditional Culture and IDEXX (MPN Method) for most bacterial groups.

Fecal *Streptococcus* shows a borderline difference (p ≈ 0.058), suggesting IDEXX may yield slightly lower values than traditional culture, but this is not conclusive at the 95% confidence level.
